# Hsa_circ_0001485 promoted osteogenic differentiation by targeting BMPR2 to activate the TGFβ-BMP pathway

**DOI:** 10.1186/s13287-022-03150-1

**Published:** 2022-09-05

**Authors:** Shan-Chuang Chen, Tao Jiang, Qi-Yu Liu, Zi-Tao Liu, Yu-Fei Su, Hai-Tao Su

**Affiliations:** 1 Department of Orthopaedics, The Second Affiliated Hospital of Guangzhou University of Chinese Medicine, No. 55 Inner Ring West Road, Guangzhou Higher Education Mega Center, Guangzhou, 510006 Guangdong China; 2 Department of Rehabilitation and Recovery, Albury Wodonga Health, Albury, NSW 2640 Australia

**Keywords:** Osteogenic differentiation, hsa_circ_0001485, TGFβ-BMP pathway, Osteoporosis

## Abstract

**Background:**

Circular RNAs (circRNAs) are a new type of stable noncoding RNA and have been proven to play a crucial role in osteoporosis. This study explored the role and mechanism of hsa_circ_0001485 in osteogenic differentiation.

**Methods:**

Kyoto Encyclopedia of Genes and Genomes (KEGG) analysis and Gene Ontology (GO) enrichment analysis were performed according to the previous sequencing data in human bone marrow mesenchymal stem cells (BMSC) before and after the induction of osteogenic differentiation on the differentially expressed circRNAs, to screen out signaling pathways associated with osteogenic differentiation. The hFOB 1.19 cells were used to verify the function and mechanism of specific circRNAs in osteogenic differentiation. Additionally, small interfering fragments and overexpression plasmids were used to determine the role of specific circRNAs during osteogenic differentiation. Furthermore, pull-down experiments and mass spectrometry were performed to determine the proteins that bind to specific circRNAs.

**Results:**

The KEGG and GO enrichment analyses showed that the TGFβ-BMP signaling pathway was related to the osteogenic differentiation process, and four circRNAs were associated with the pathway. The quantitative polymerase chain reaction analysis revealed that hsa_circ_0001485 expression was increased during the osteogenic differentiation process of BMSCs. Knockdown of hsa_circ_0001485 suppressed the activity of the alkaline phosphatase enzyme and the expression of RUNX2, osteopontin, and osteocalcin in the osteogenic hFOB 1.19 cells, whereas overexpression of hsa_circ_0001485 promoted their expression. Additionally, we found that hsa_circ_0001485 and BMPR2 targeted binding to activate the TGFβ-BMP signaling pathway and promoted osteogenic differentiation through mass spectrometry analysis.

**Conclusion:**

This study demonstrates that hsa_circ_0001485 is highly expressed in the osteogenic hFOB 1.19 cells, which activate the TGFβ-BMP pathway through targeted binding of BMPR2, and plays a positive role in regulating osteogenic differentiation.

## Introduction

Osteoporosis (OP) is a systemic bone disease in which bone density and mass decrease, leading to fractures [1, 2]. With the rapid improvement in the social economy, the aging of the population is developing rapidly, increasing the incidence of chronic diseases related to aging, such as osteoporosis, causing a heavy economic burden [3]. More seriously, osteoporotic fractures are closely related to the high risk of subsequent fractures and mortality [4]. However, the etiology and pathogenesis of OP are not fully understood.

Studies have manifested that the main pathogenesis is the disruption of the coupling balance between osteoblast-mediated bone formation and osteoclast-mediated bone resorption, resulting in more significant bone resorption than bone formation [5, 6]. Therefore, promoting osteoblast proliferation and differentiation can effectively prevent and treat OP. The BMSCs, as a class of multipotential stem cells, are the precursor cells of osteoblasts and can differentiate into osteoblasts under certain induction conditions [7, 8]. Several studies have also revealed that BMSCs play a key role in OP. For example, Let-7a-5p attenuates osteogenesis of BMSCs in postmenopausal OP mice [7]; downregulation of TP53INP2 prevents osteogenic differentiation of BMSCs during OP [9]; the miR-291a-3p accelerates osteogenic differentiation of BMSCs in dexamethasone-induced OP [10]; the SIRT3 affects BMSCs senescence and senescence OP [11]. Therefore, further investigation of the molecular mechanisms involving the differentiation of BMSCs into osteoblasts will provide new insights into preventing OP and its therapy.

Circular RNA (circRNA) is a noncoding RNA molecule with a closed-loop structure that is stable in expression, not easily degraded, and widely exists in various eukaryotic organisms [12, 13]. The circRNAs were shown to play an influential role in biological growth and development, disease occurrence and development through miRNA sponging, protein binding, and gene transcription and coding functions [14–16]. Recently, researchers have paid attention to the role of circRNAs in osteogenic differentiation [17]. The decreased bone formation ability of the bone marrow mesenchymal stem cells (BMSCs) is one of the major causes of OP. However, the specific mechanism of BMSCs in osteoblast differentiation is unclear. Studies have revealed that hsa_circ_0006215 competitively binds to miR-942-5p and promotes the differentiation of BMSCs and osteogenesis–angiogenesis coupling by regulating RUNX2 [18]. The RUNX2 is an essential molecule in osteodifferentiation and bone development and is responsible for activating osteoblast differentiation marker genes [19].

Additionally, osteopontin (OPN), alkaline phosphatase (ALP), and osteocalcin (OCN) are the most investigated osteogenic-related genes [20]. Liu et al. found that circRNA AFF4 activated the SMAD1/5 signaling pathway by binding to miR-135a-5p and played a role in the bone differentiation of BMSCs [21]. Han et al. revealed that hsa_circ_0076690 was significantly correlated with bone mineral density (BMD) in patients with OP, with a sensitivity of 79% and a specificity of 85%, and can be used as a potential diagnostic biomarker of OP [22]. Moreover, hsa_circ_0024097 acts as a ceRNA and sponges with miR-376b-3p targeting Yap1 and passes through the Wnt/β-catenin pathway to promote osteogenic differentiation [23]. Although the effect of circRNA on osteogenic differentiation has become a hot topic for researchers, it has only revealed the tip of the iceberg. The sponge mechanism has been used in many previous investigations to explain the role of circRNA in osteogenic differentiation since circRNA functions like another noncoding LncRNA, which can affect different physiological and pathological conditions by directly binding to proteins [24, 25]. However, further elucidation of whether circRNA can directly bind to essential osteogenic proteins to affect osteogenic differentiation still lacks research.

In this study, the Kyoto Encyclopedia of Genes and Genomes (KEGG) and Gene Ontology (GO) enrichment analysis of differentially expressed circRNAs showed that the TGFβ-BMP signaling pathway was involved in the osteogenic differentiation process, in which hsa_circ_0001485 played an indispensable role. The TGFβ-BMP signaling pathway was proven to play a vital function in bone development and postnatal bone homeostasis. Furthermore, it has clinical significance for treating osteoporosis, fracture healing, and osteoarthritis [26]. We also applied mass spectrometry (MS) to analyze the target proteins associated with hsa_circ_0001485 and TGFβ-BMP pathway. We also discovered that hsa_circ_0001485 might promote osteogenic differentiation by activating the TGFβ-BMP pathway through targeted binding with the BMPR2 protein. This study revealed a new mechanism by which circRNAs regulate osteogenic differentiation, laying a foundation for targeted therapy of OP.

## Methods and materials

### Cell culture

The BMSCs (SALIAI, Guangzhou, China; G02001), MG63 (CRL-1427, ATCC), and hFOB 1.19 (Cellcook, Guangzhou, China; CC4005) were incubated in DMEM/F12 (GIBCO; A4192001) supplemented 10% fetal bovine serum (FBS; GIBCO, 10099141C) in a 5% CO_2_ incubator at 37 °C. When the cell density reached approximately 80%, they were dissociated with trypsin and inoculated into 6-well plates for other experiments.

### BMSCs-induced osteogenesis

The BMSCs were inoculated into 6-well plates and cultured to adhere to the wall. Osteogenic differentiation of BMSCs was induced using an osteoblast differentiation kit (#HuxMA-90021, Cyagen, Guangzhou, China) according to the instructions [27], and the cells were collected at 0, 7, 14, and 21 days of induction. The medium was changed once in 3 days. The kit included OriCell®Basal Medium For Cell Culture (177 mL; BLDM-03011), OriCell®Fetal Bovine Serum (Superior-Quality) (20 mL; FBSSR-01021), and OriCell®Supplement For Human BMSC Osteogenic Differentiation (3 mL; HUXMX-04021).

### Cell transfection and infection

We designed hsa_circ_0001485 interference fragments (AACAGTTTCAGAGTGGCAGAA) to suppress the expression of hsa_circ_0001485 and synthesized them using GenePharma Co. Ltd. (Shanghai, China). First, serum-free medium (DMEM/F12) and interference fragments were successively added to the transfection tube and thoroughly mixed. Subsequently, the transfection agent, Lipofectamine 3000 (Invitrogen, Carlsbad, CA, USA), was added to the same tube. After thorough mixing, the mixture was left to stand for 10–15 min. Next, the mixture was added to the culture plate and replaced after 48 h with a medium containing 10% FBS. Additionally, for the hsa_circ_0001485 overexpression, hsa_circ_0001485 overexpression plasmids were synthesized by RiboBio (Guangzhou, China) and transfected into hFOB 1.19 cells for overexpressing hsa_circ_0001485 (OE-circ1485), and the vector LV003 kindly provided by Forevergen (Guangzhou, China) was used as a negative control. Finally, the transfection efficiency was measured by quantitative polymerase chain reaction (qPCR), and cells were collected for subsequent experiments.

Moreover, Guangzhou Forevergen Bioscience Company further constructed hsa_circ_0001485 knockdown and overexpression lentiviruses and their vector controls (NC1, Sh-hsa_circ_0001485, NC2, and OE-hsa_circ_0001485). The sequence for knockdown was transformed from the siRNA sequence, which was 5′-AACAGTTTCAGAGTGGCAGAA-3′, and the corresponding NC1 sequence was 5′-TTCTCCGAACGTGTCACGTTTC-3′. Lentiviruses (10^9^ TU/mL) and polybrene (5 μg/mL, Sigma) were added to the medium and incubated with BMSCs together for 24 h at a multiplicity of infection of 30. The qPCR measured the transfection efficiency.

### Quantitative real-time polymerase chain reaction

Total RNA was extracted using TRIzol reagent (Invitrogen, TR118-500) according to the manufacturer’s instructions. Reverse transcriptase reactions were performed using the Prime Script RT kit (Takara, Japan, RR047A). The reverse transcription polymerase chain reaction (RT–PCR) was performed using qPCR and SYBR Premix Ex Taq TM (Takara, Japan, RR82WR) and an ABI 7500 sequence detection polymerase chain reaction (PCR) system (Applied Biosystems, USA). Different primers were designed to identify circRNA expression (hsa_circ_0006952, hsa_circ_0008532, hsa_circ_0005564, and hsa_circ_0001485). Convergence and divergence primers for hsa_circ_0001485 and GAPDH were used to verify the circRNA circular formed using agarose gel electrophoresis, and then, the divergence primers were used to perform Sanger sequencing for hsa_circ_000148 products. The GAPDH was used as an internal control to normalize the levels of circRNAs. Table 1 presents the primer sequences.

**Table 1:** Primer sequence of this study

Primer name	Sequence	Product length
circ1485-DF	5′ TCACGCATGTCAAATTCTCATAC 3′	144 bp
circ1485-DR	5′ TTGTTTCCTCAGGGCTATATGG 3′	
GAPDH-CF	5′ GAGTCAACGGATTTGGTCGT 3′	185 bp
GAPDH-CR	5′ GACAAGCTTCCCGTTCTCAG 3′	
circ6952-F	5′ TAGACAGAGAAGCTGGGCGTG 3′	170 bp
circ6952-R	5′ GTGGATGCTGGATGGTTTGAA 3′	
circ1085-F	5′ GAAAGAGAAAGTGGAGATCGAA 3′	201 bp
circ1085-R	5′ TCATCAATGTGTGAGGTAAAAGAC 3′	
circ7386-F	5′ ACTGTGGAATGCCCTCCTGTT 3′	116 bp
circ7386-R	5′ AATCTGGCTTCTCTTCTTGTTGG 3′	
circ8532-F	5′ ATGAAAACACAGAGCTGAGGAAA 3′	172 bp
circ8532-R	5′ TGAGAAATGGAATCACAAAAGGA 3′	
circ5564-F	5′ CCAGTGGCTAAAGCACATCG 3′	125 bp
circ5564-R	5′ CAGAGGGCACCACAGAGTCC 3′	
BMPR2-F	AAATAGCCTGGCAGTGAG	196 bp
BMPR2-R	ATGTGACAGGTTGCGTTC	
circ1485-CF	CCCAGACAATAAATACCG	193 bp
circ1485-CR	GCTACGCCTACTGTGATA	
GAPDH-DF	TCCTCACAGTTGCCATGTAGACCC	
GAPDH-DR	TGCGGGCTCAATTTATAGAAACCGGG	

### Alizarin red (AR) staining

The method of AR staining has been described previously [28]. First, the cell culture medium was removed and washed with PBS twice. Subsequently, 2 ml of 4% formaldehyde was added and fixed for 30 min. Next, the cells were washed twice with PBS, and 1 ml of 0.2% AR solution (Solarbio, China, G1450) was added to each well for staining for 5 min. After washing with PBS, the images of osteogenesis staining were captured under a microscope. Subsequently, the samples were treated with 1 ml of 10% cetylpyridinium chloride (CPC; Sigma) for 30 min to elute the stain. Finally, 100 μL of the eluted stain was added to a 96-well plate and tested using a spectrophotometer at 550 nm. The calcium content of each sample was analyzed based on the standard curve prepared by ALZ and CPC.

### ALP staining

The ALP staining was performed to test the osteogenic ability of the cells. The osteogenic hFOB 1.19 cells were washed with PBS, fixed with 10% formaldehyde for 20 min, and washed with double steaming water twice. The ALP staining (Sigma–Aldrich, C3206) was added and placed in a horizontal shaker for incubation for 1 h in the dark. The cells were rinsed with double steaming water four times for 5 min. The excess double steam water was removed, and the cells were observed under an inverted microscope and photographed for analysis.

### ALP enzyme activity determination

The osteogenic hFOB 1.19 cells were rinsed with PBS three times and lysed with RIPA lysis buffer (Beyotime), and the supernatant was collected and added to 96-well plates. Reagent from the ALP Assay kit (Beyotime, China, P0321S) was added to a 96-well plate. After incubation at 37 °C for 30 min, absorbance was measured at 405 nm, and the ALP activity was assessed according to the instructions.

### Western blot analysis

In the presence of a protease inhibitor (Beyotime, China, P1005), the osteogenic hFOB 1.19 cells were lysed using RIPA lysis buffer (Beyotime, China, P0013B), and centrifugation at 4 °C (12,000 rpm) for 15 min collected the supernatant. Protein samples were separated by SDS–PAGE electrophoresis and then transferred to polyvinylidene fluoride (PVDF; Millipore, IPVH20200) membranes. The TBST containing 5% skim milk (Beyotime, China, P0216) was used to block the nonspecific binding site for 1 h, and the antibodies were incubated overnight at 4 °C. After incubation with the antimouse IgG (Jackson, 115-035-003) or antirabbit IgG (Jackson, 111-035-003) at room temperature for 1 h, the signal was detected with an enhanced chemiluminescence reagent (Beyotime, China, P0018FM). Table 2 presents the list of the primary antibodies.

**Table 2:** The primary antibody information of this study

Primary antibody name	Company and catalog	Dilution	Molecular weight (kDa)
RUNX2	CST, 8486	0.736111111	55–62
OPN	abcom,ab8448	0.736111111	66
OCN	Abcam, ab133612	0.736111111	11
GAPDH	Proteintech 60,004–1-Ig	5.597222222	36

### RNA pull-down and silver staining

The CircRNA pull-down was performed using the Magnetic RNA–protein pull-down kit (ThermoFisher, 20164, USA), as previously reported [29]. The osteogenic hFOB 1.19 cells transfected with hsa_circ_0001485 overexpression plasmids were lysed and hybridized with the probe and incubated at 70 °C for 5 min. Streptavidin magnetic beads were then incubated with the beads at room temperature for 30 min. Next, the protein binding buffer and total protein were added to a tube containing streptavidin magnetic beads. After rotary incubation at 4 °C for 1.5 h, streptavidin magnetic beads were washed thrice with washing buffer and then incubated with the elution buffer at 37 °C for 15 min. After the RNA was pulled down, the same amount of protein was added to the 10% polypropylene gel. After electrophoresis for 1.5 h, silver staining (Beyotime, China, P0017S) was performed according to the instructions.

### MS analysis

The differential bands displayed by silver staining were cut off for MS analysis. The protein bands were cut out and washed using Milli-Q water for 1 min. Next, the protein bands were added to the decolorization solution in a 37 °C constant temperature box for 30 min. After centrifugation, the supernatant was added with 100% ACN and shaken for 30 s until the colloidal particles turned white. Next, the collected liquid was added to 25 mM DTT/50 mM NH_4_HCO_3_ and reacted at 56 °C for 30 min. Subsequently, DTT was collected and treated with 55 mM IAA/50 mM NH_4_HCO_3_ in the darkness for 30 min. After three washes, the sample was dehydrated with 100% ACN until colloidal particles turned white. After enzyme digestion, the peptide was purified, and the sample was dissolved in solution (0.1% formic acid, 2% acetonitrile). After centrifugation (13,000 rpm at 4 °C for 20 min), the supernatant was collected and identified through MS. The MS analysis was conducted using a nano-ultra‐performance liquid chromatography (nanoUPLC) system (Waters, Milford, MA, USA) and Synapt High Definition Mass Spectrometry (Waters, Milford, MA, USA), which was similar to the previous study [30].

### Bone formation of BMSCs in vivo

The BMSCs infected with lentiviruses (NC1, Sh-hsa_circ_0001485, NC2, and OE-hsa_circ_0001485) were cultured in osteoblast differentiation solution for 7 days before the in vivo study. After being trypsinized and resuspended directly in DMEM (Thermo Fisher Scientific, C11965500BT), BMSCs (5 × 10^5^) were loaded onto 40 mg hydroxyapatite/tricalcium phosphate (HA/TCP; Zimmer) and then implanted into the dorsal subcutaneous space on the two symmetrical sites of 8-week-old BALB/c-nu/nu female mice (Guangzhou Forevergen Bioscience Company; *n* = 5 per group). Specimens were harvested 8 weeks after implantation, and the animals were euthanized by CO_2_ asphyxiation. The bone constructs were fixed in 4% paraformaldehyde and then decalcified for 10 days in 10% EDTA (pH 7.4). After decalcification, the specimens were dehydrated and subsequently embedded in paraffin. Sections (5 mm thickness) were stained with hematoxylin, eosin (H&E), and Masson’s trichrome staining according to the standard protocol. The ethical committee approved our experiment with Guangzhou Forevergen Biosciences (Guangzhou, China; Approval No. IACUC-AEWC-F2010008).

### Statistical analysis

Data are presented as the mean ± standard deviation. Statistical analysis was performed using GraphPad Prism 8.0 (GraphPad Software, La Jolla, CA, USA) and SPSS software Version 22.0 (IBM Corporation, New York, USA). All procedures were repeated thrice. A student’s *t* test was performed to test for statistical significance between two groups; three or more groups were compared using one-way analysis of variance. A value of *P* < 0.05 and *P* < 0.01 was considered statistically significant.

## Results

### Hsa_circ_0001485 was upregulated in the osteogenic differentiation of BMSCs

**Fig. 1:**
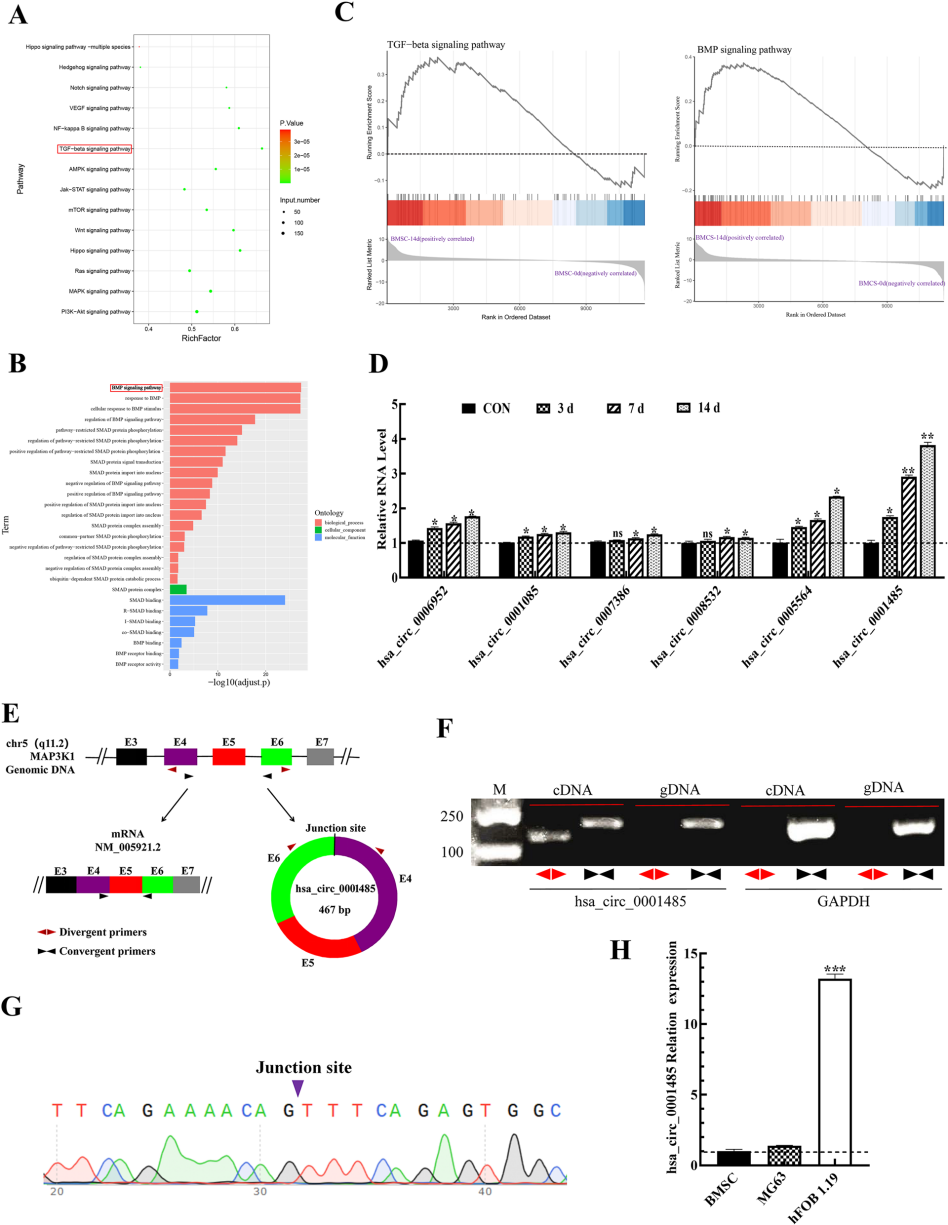
Expressions of hsa_circ_0001485 in BMSC osteogenic differentiation. **A** KEGG analysis of differentially expressed circRNA host genes in the BMSC-14d group. **B** GO enrichment analysis of differentially expressed circRNA host genes in the BMSC-14d group. **C** Analysis of TGFβ and BMP pathways using GSEA. **D** qPCR was used to verify the circRNA expression of BMSCs at 0, 3, 7, and 14 d of induction. **E** The structure of hsa_circ_0001485. **F** The agarose gel electrophoresis detects the form of hsa_circ_0001485. **G** Sanger sequencing results of hsa_circ_0001485, in which the arrow indicates the cyclization site. **H** qPCR detect has the expression of hsa_circ_0001485 in BMSCs and MG63 and hFOB 1.19 cells. The results are representative data from three replicates, and the data mean ± *SD* (ns, no statistical significance; *P < 0.05; **P < 0.01; ***P < 0.001)

We induced osteogenic differentiation of BMSCs to investigate the function of circRNAs in the occurrence and development of OP. Next, we examined the differentially expressed circRNAs between the induced and control groups through RNA sequencing. In previous sequencing data, there were 3440 circRNAs in the control group and 3893 circRNAs in the BMSC-14d group, among which 2191 circRNAs were specific to the BMSC-14d group [31]. The KEGG and GO analyses were performed to conduct a bioenrichment analysis of the differential circRNAs unique to the BMSC-14d group. The signaling pathways related to osteogenic differentiation were screened, from which the TGFβ-BMP signaling pathway was selected as the object of exploration (Fig. 1A, B). The GSEA of the TGFβ and BMP signaling pathways showed that both were activated at BMSC-14d compared with BMSC-0d (Fig. 1C). Subsequently, we selected four relevant circRNAs from the TGFβ-BMP signaling pathway, hsa_circ_0006952, hsa_circ_0008532, hsa_circ_0005564, and hsa_circ_0001485 (Table 3), and detected their expressions in BMSCs at 0, 3, 7, and 14 d by qPCR. The data showed that relative to the control (BMSC-0d) group, the level of hsa_circ_0001485 was significantly upregulated in BMSC osteogenic differentiation groups, especially during the induction of 14 days (Fig. 1D), so hsa_circ_0001485 was used as the target circRNA for research. Figure 1E shows the structure of hsa_circ_0001485, located on chromosome 5, MAP3K1, and exons 4–6. The PCR production of cDNA and gDNA demonstrated that the divergent primer circ_0001485 could amplify the loop in cDNA from hFOB1.19 cells (Fig. 1F). Then, the amplified product of the divergent primer in cDNA was sequenced by Sanger sequencing, and the cyclic site was identified to confirm the cyclic structure of hsa_circ_0001485 (Fig. 1G). We investigated the expression of hsa_circ_0001485 in two human osteoblasts (MG63 cells and hFOB 1.19 cells), and the results revealed that hsa_circ_0001485 was significantly increased in hFOB cells compared with undifferentiated BMSC and MG63 cells (Fig. 1H), so hFOB 1.19 cells were used for the function and mechanism of hsa_circ_0001485 in osteogenic differentiation.

**Table 3:** The selected circRNAs expression in RNA seq

circBase_ID	BMSC_CON-TPM	BMSC_14d-TPM	Regulation	*p* value
hsa_circ_0006952 [32]	0.001	74.33	Up	0.302662
hsa_circ_0008532 [33]	0.001	74.33	Up	0.302662
hsa_circ_0005564 [34]	0.001	148.66	Up	0.0859496
hsa_circ_0001485 [35]	0.001	74.33	Up	0.302662

### Hsa_circ_0001485 facilitated osteogenic differentiation in vitro

**Fig. 2:**
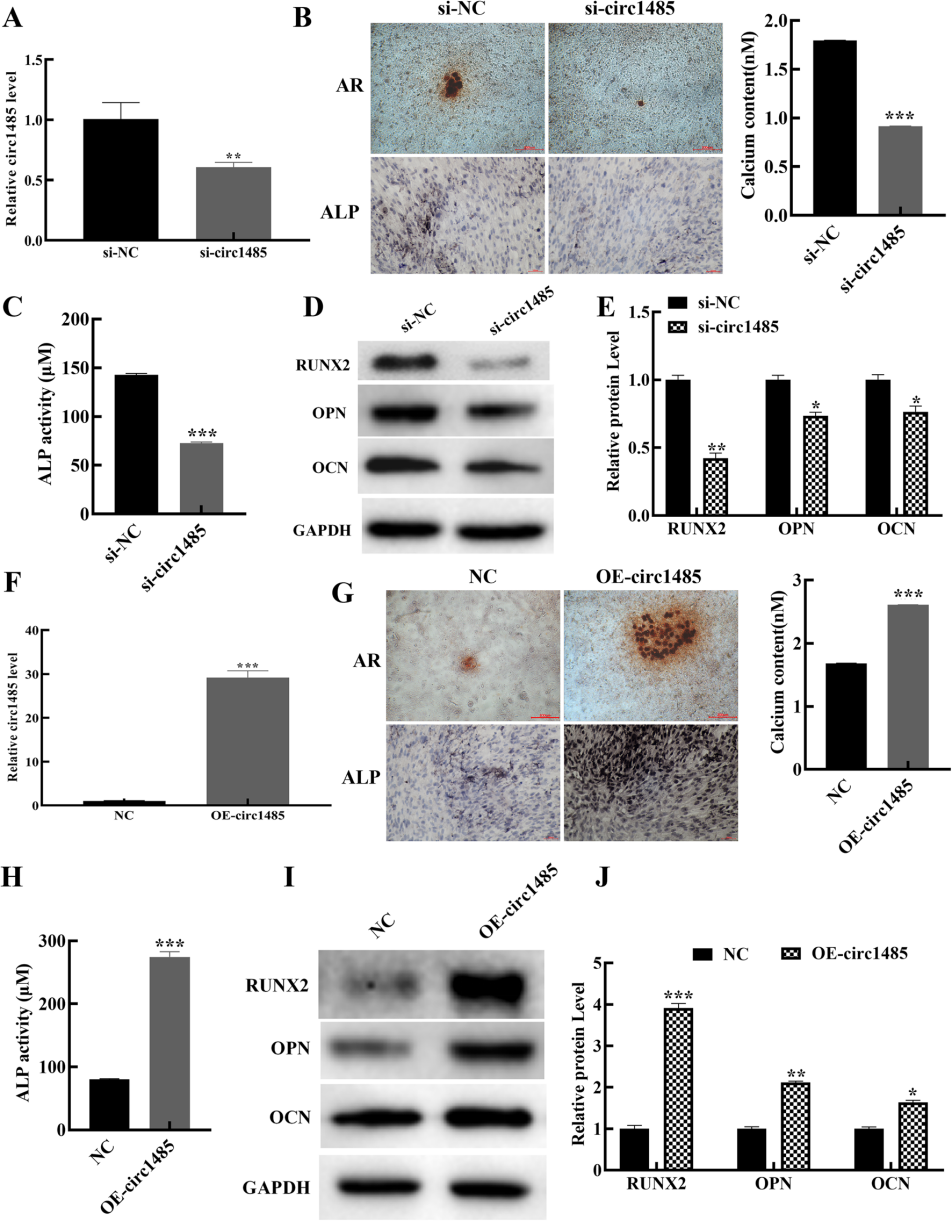
Hsa_circ_0001485 promoted osteogenic differentiation in the osteogenic hFOB 1.19 cells. **A** qPCR evaluated efficiency of hsa_circ_0001485-targeted siRNA (si-circ1485) after 48 h siRNA transfection in hFOB 1.19 cells. **B** Alizarin red (AR) and ALP staining of osteogenic hFOB 1.19 cells transfected with si-circ1485. For AR, magnification, 100 ×, scale bar = 200 μm; for ALP, magnification, 200 ×, scale bar = 100 μm. **C** ALP activity assay in osteogenic hFOB 1.19 cells transfected with si-circ1485. **D**, **E** The protein levels of RUNX2, OPN, and OCN were detected via western blot after transfection with si-circ1485 and quantification by densitometric analysis. **F** Osteogenic hFOB 1.19 cells overexpressing hsa_circ_0001485 (OE-circ1485) were constructed by plasmid and confirmed by qPCR assay. **G** AR staining and ALP staining of osteogenic hFOB 1.19 cells transfected with OE-circ1485. For AR, magnification, 100 × , scale bar = 200 μm; for ALP, magnification, 200 ×, scale bar = 100 μm. . **H** ALP activity assay in cells transfected with OE-circ1485. **I**, **J** After transfection with OE-circ1485 and quantification by densitometric analysis, Western blot detected RUNX2, OPN, and OCN levels. The results are representative data from three replicates, and the data mean ± *SD* (*P < 0.05; **P < 0.01; ***P < 0.001)

The hFOB 1.19 cells were transfected with hsa_circ_0001485 small interfering RNA (si-circ1485) for 48 h. Subsequently, hFOB 1.19 cells were cultured in a differentiation medium to induce osteogenic differentiation for 14 days. The data revealed that the expression of hsa_circ_0001485 was potently suppressed by hsa_circ_0001485 silencing in the osteogenic hFOB 1.19 cells (Fig. 2A). Through AR and ALR staining, we found that the number of calcified nodules and ALP activity in the osteogenic hFOB 1.19 cells was reduced in the si-circ1485 group compared with that in the si-NC group (Fig. 2B, C). Meanwhile, we also disclosed that relative to the si-NC transfection group, transfection of si-circ1485 could dramatically attenuate the expressions of RUNX2, OPN, and OCN proteins in the osteogenic hFOB 1.19 cells (Fig. 2D, E). Then, we constructed the hsa_circ_0001485 overexpression vector (OE-circ1485) and transfected the osteogenic hFOB 1.19 cells. We discovered that the expression level of hsa_circ_0001485 in the overexpression group was higher than that in the control (NC) group (Fig. 2F). Besides, we proved that hsa_circ_0001485 overexpression could potently increase the number of calcified nodules and the activity of ALP in the osteogenic hFOB 1.19 cells (Fig. 2G, H). Consistent with expectations, hsa_circ_0001485 overexpression also markedly enhanced the expression levels of RUNX2, OPN, and OCN proteins in the osteogenic hFOB 1.19 cells (Fig. 2I, J). Based on the above results, it was shown that hsa_circ_0001485 could induce osteogenic differentiation in hFOB 1.19 cells in vitro*.*

### Hsa_circ_0001485 activated the TGFβ-BMP signaling pathway by combining with BMPR2 in the osteogenic hFOB 1.19 cells

**Fig. 3:**
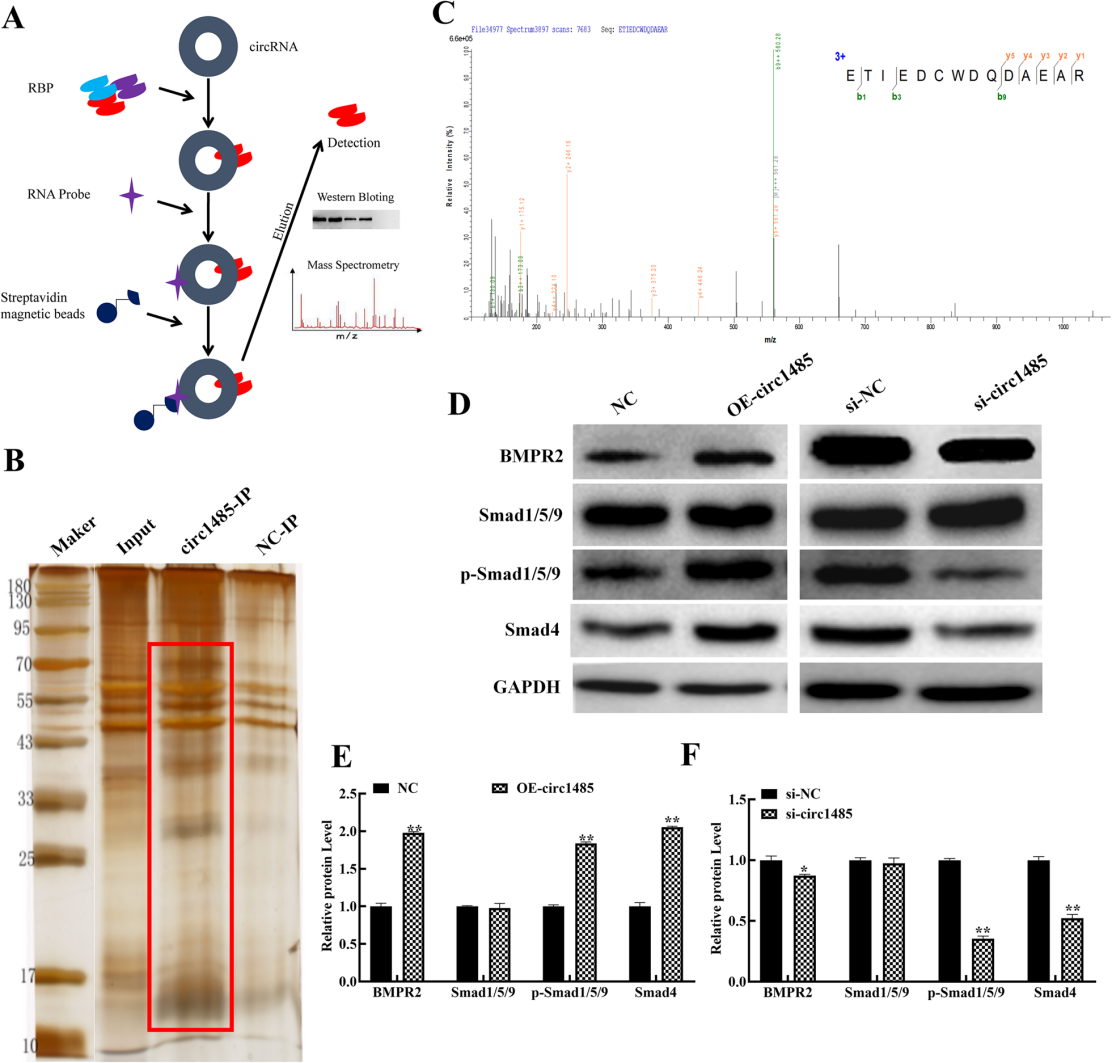
Hsa_circ_0001485 bound to BMPR2 to activate the BMP signaling pathway in the osteogenic hFOB 1.19 cells. **A** Design of the pattern diagram of the hsa_circ_0001485 probe for the pull-down test. **B** Silver staining analyzed the pull-down products. **C** The target protein BMPR2 was identified by mass spectrometry. **D** The western blot detected the expression of BMPR2 and its downstream proteins Smad1/5/9, p-Smad1/5/9, and Smad4. **E, F** Quantitative analysis of BMPR2, Smad1/5/9, p-Smad1/5/9, and Smad4 was performed by densitometry. The results represent data from three replicates, and the mean ± SD (**P < 0.01)

To confirm the function of hsa_circ_0001485 in the TGFβ-BMP signaling pathway, we designed a probe to conduct a pull-down experiment on osteogenic hFOB 1.19 cells transfected with OE-1485 (Fig. 3A). A silver staining test was performed on the pull-down products. Differences were found between the experimental probe group (circ1485) and the control probe group (NC; Fig. 3B). We adopted the gelatinization method. We cut the parts with apparent differences in bands for MS analysis. The MS identified 523 proteins, and we noticed a target protein, BMPR2 (the green and orange peaks are the detected BMPR2 peaks), which played an important role in BMP signaling pathway activation (Fig. 3C). As a major type of BMP receptor, BMPR2 combines with multiple BMPs for the downstream BMP signaling pathway activation [36]. We measured the expression of downstream proteins in the BMP signaling pathway after transfection of OE-circ1485 and si-circ1485. The results confirmed that the expression of BMPR2, p-Smad1/5/9 and Smad4, was markedly increased in the hsa_circ_0001485 overexpression group relative to that in the NC group, and these protein levels were dramatically decreased in hsa_circ_0001485 silencing group comparable to that in the si-NC group (Fig. 3D–F).

### Hsa_circ_0001485 promoted osteogenic differentiation through the TGFβ-BMP signaling pathway in the osteogenic hFOB1.19 cells

**Fig. 4:**
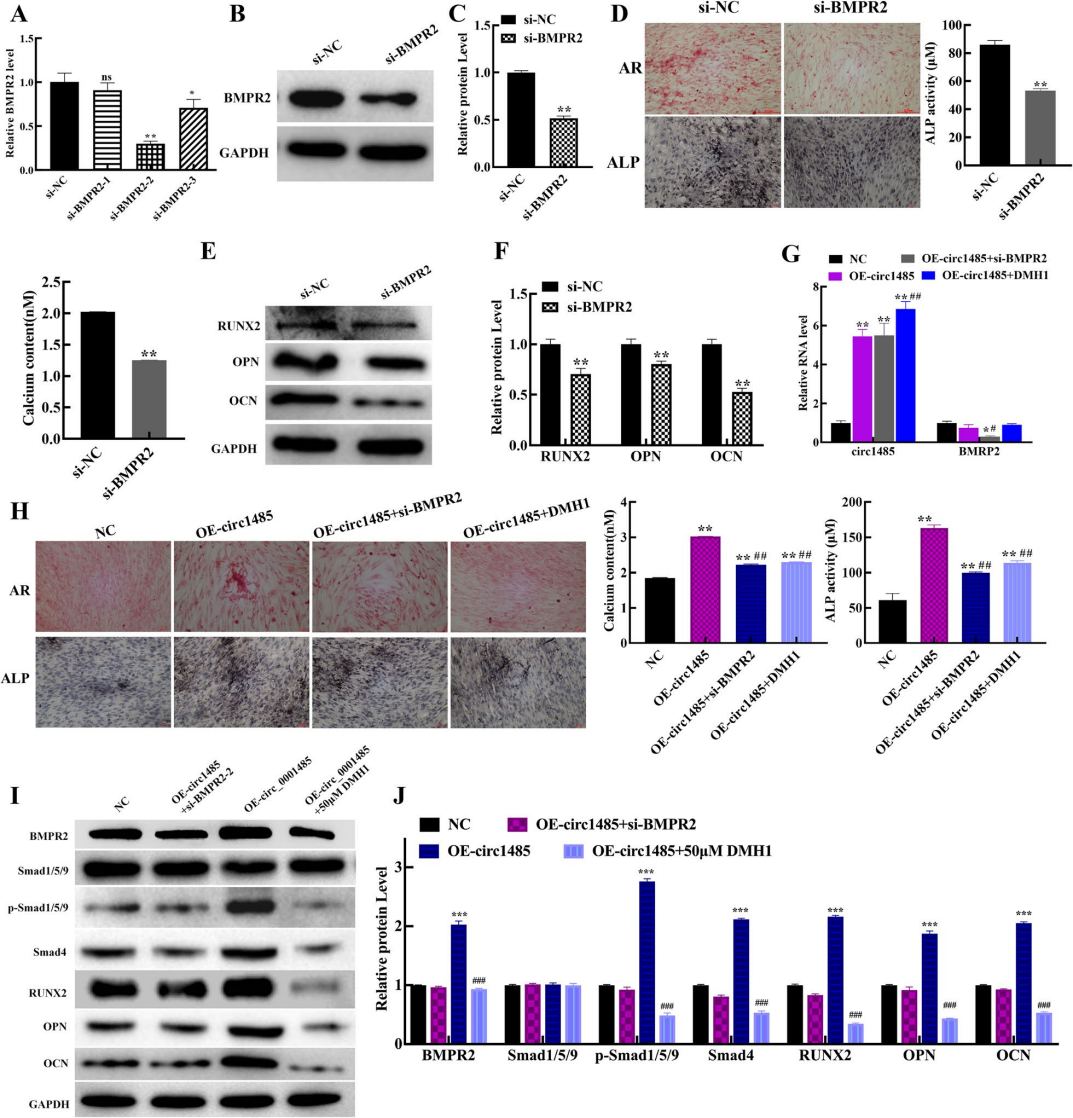
Hsa_circ_0001485 affected osteogenic differentiation through the TGFβ-BMP pathway in the osteogenic hFOB 1.19 cells. **A** qPCR determined the knockdown efficiency of three siRNAs targeting BMPR2. **B**, **C** Western blot analysis of the protein expression of BMPR2 in osteogenic hFOB 1.19 cells transfected with si-BMPR2 and quantification by densitometric analysis. **D** AR and ALP staining was performed on the cells transfected with si-BMPR2. For AR, magnification, 100 × , scale bar = 200 μm; for ALP, magnification, 200 × , scale bar = 100 μm. **E**, **F** The expression levels of RUNX2, OPN, and OCN in cells transfected with si-BMPR2 were detected using western blot and quantitative analysis. **G** Osteogenic hFOB 1.19 cells were transfected with OE-circ1485 and then transfected with si-BMPR2 or DMH1 inhibitor, and qPCR measured the expression of hsa_circ_0001485 and BMPR2. **H** AR and ALP staining was performed on cells transfected with OE-circ1485 and then transfected with si-BMPR2 or DMH1 inhibitor. For AR, magnification, 100 ×, scale bar = 200 μm; for ALP, magnification, 200 ×, scale bar = 100 μm. **I**, **J** Osteogenic hFOB 1.19 cells were transfected with OE-circ1485 and then transfected with si-BMPR2 or DMH1 inhibitor. Western blot analysis of the levels of BMPR2, Smad1/5/9, p-Smad1/5/9, Smad4, RUNX2, OPN, and OCN in osteogenic hFOB 1.19 cells and quantification of western blot bands by densitometric analysis. The results are representative data from three replicates, and the data mean ± *SD* (*P < 0.05; **P < 0.01; ***P < 0.001 vs. NC group; ^#^*P* < 0.05; ^##^*P* < 0.01; ^###^*P* < 0.001 vs. OE-circ1485 group)

To further query the potential function of BMPR2, we designed three interference fragments of BMPR2. After transfection for 48 h in the osteogenic hFOB 1.19 cells, we found that si-BMPR transfection had strong interference effects, especially si-BMPR2-2 (Fig. 4A). Western blot data revealed that the protein expression level of BMPR2 was notably decreased in the BMPR2 silencing group compared with the si-NC group, further indicating the effectiveness of BMPR2-2 interference in the osteogenic hFOB1.19 cells (Fig. 4B, C). Through ALP and AR staining, it was observed that after interference with BMPR2, the number of calcified nodules and the expression of ALP in the osteogenic hFOB 1.19 cells decreased (Fig. 4D). Additionally, the protein expression of RUNX2, OPN, and OCN was observably attenuated in the si-BMPR2 group relative to that in the si-NC group (Fig. 4E, F), indicating that interfering with BMPR2 suppressed the osteogenic differentiation ability of the osteogenic hFOB 1.19 cells. We further transfected OE-circ1485 into the hFOB 1.19 cells, which were then processed as si-BMPR2 or BMPR2 inhibitor (DMH1) simultaneously to inhibit the expression of BMPR2. The results denoted that after osteogenic induction, hsa_circ_0001485 overexpression significantly upregulated hsa_circ_0001485, and the upregulation of hsa_circ_0001485 expression mediated by hsa_circ_0001485 overexpression was not modulated by BMPR2 silencing, indicating that BMPR2 is a downstream regulatory gene of hsa_circ_0001485. We also discovered that hsa_circ_0001485 overexpression did not affect the mRNA level of BMPR2, suggesting that hsa_circ_0001485 only affected the protein level of BMPR2. (Fig. 4G). Besides, overexpression of hsa_circ_0001485 prominently promoted the number of calcified nodules and the expression of ALP, while the promotion mediated by hsa_circ_0001485 overexpression could also be memorably inhibited by si-BMPR2 or DMH1 (Fig. 4H). Moreover, we discovered that inhibition of BMPR2 notably weakened the increase in BMPR2, p-Smad1/5/9, Smad4, RUNX2, OPN, and OCN expressions induced by hsa_circ_0001485 overexpression (Fig. 4I, J), which indicated that hsa_circ_0001485 facilitated osteogenic differentiation through the TGFβ-BMP pathway.

### Hsa_circ_0001485 facilitated osteogenic differentiation in vivo

**Fig. 5:**
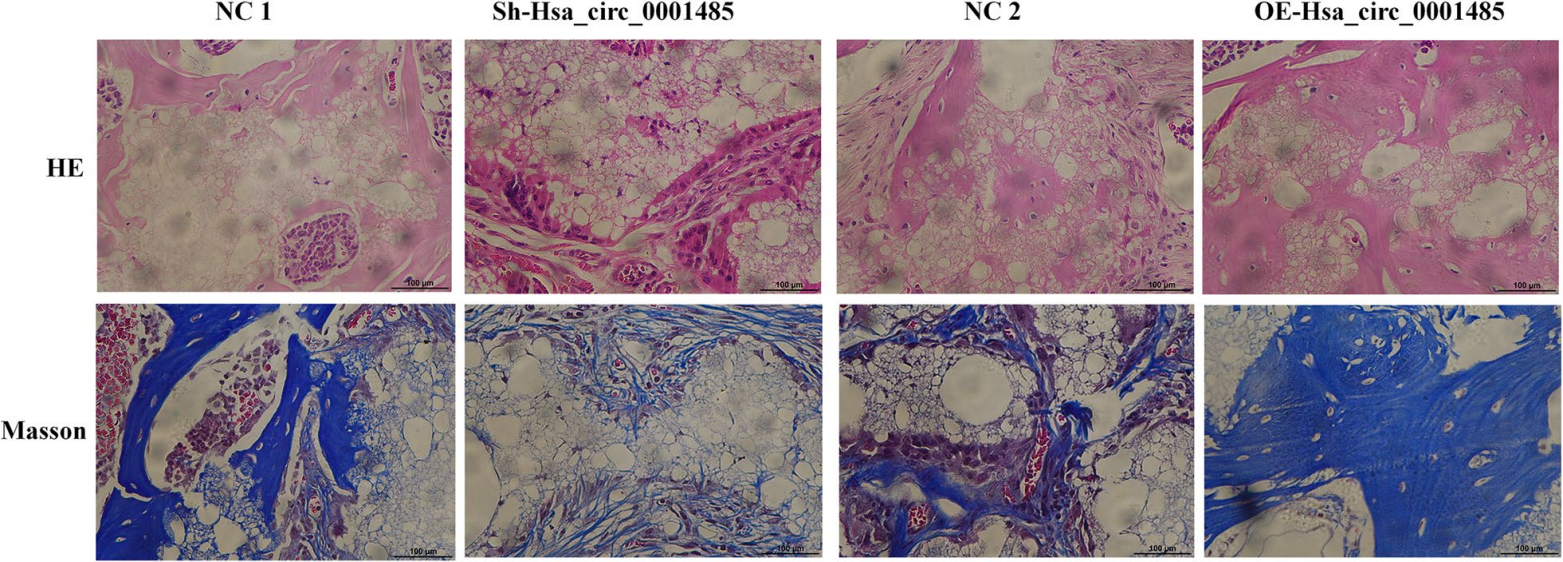
Hsa_circ_0001485 facilitated osteogenic differentiation in vivo*.* Compared with the control group, H&E staining and Masson staining of MSCs with HA/TCP in the hsa_circ_0001485 knockdown or overexpression group. Scale bar, 100 µm; *n* = 5

Finally, we constructed a classic bone formation model in vivo to determine the role of hsa_circ_0001485 in osteoblast differentiation. The BMSCs infected with lentiviruses (NC1, sh-hsa_circ_0001485, NC2, and OE-hsa_circ_0001485) were cultured in an osteogenic induction medium for 7 days before the in vivo study. The BMSCs were then loaded onto scaffolds and implanted into the subcutaneous space of nude mice. In the H&E and Masson staining results, the new bone formation ability was significantly attenuated in the sh-hsa_circ_0001485 group relative to that in the NC1 group. Simultaneously, the new bone formation ability was enhanced considerably in the hsa_circ_0001485 overexpression group comparable to that in the NC2 group (Fig. 5).

## Discussion

As the aging of the population increases, the size of the OP population increases, and it gradually becomes a clinical disease with a large probability of occurrence and poor treatment effects [2, 37]. Multiple signaling pathways modulate osteoblast and osteoclast differentiation and formation. Developing high-throughput sequencing technology provides strong technical support for studying complex diseases, such as osteoporosis. The CircRNA is widely expressed in human tissues and mediates various physiological events, such as organogenesis, tumorigenesis, and organ development [38]. Recent studies have confirmed that the expression profile of circRNAs is changed during the formation of osteoclasts [39]. Besides, circRNA plays a key role in the formation of osteoblasts and osteoclasts and the differentiation of BMSCs through various pathways, thus affecting the bone formation and bone resorption [40]. Also, multiple studies have proved that circRNA plays a crucial role in OP [41–43]. In our previous sequencing results, 219 unique circRNAs were identified after osteogenic differentiation of BMSCs [43]. This study performed KEGG and GO analyses to identify the pathways related to bone differentiation. The TGFβ and BMP were considered most closely related to osteogenic differentiation. Focusing on the TGFβ-BMP pathway, hsa_circ_0001485 was the most significantly increased differentially expressed circRNA.

In our study, we first used human primary BMSC cells for sequencing because the cells are closer to the actual human condition. However, due to the great difficulty of primary cell culture, the limited number of passages, and the high cost, we chose human osteogenic-related cells (MG63 and hFOB 1.19 cells) for our functional study. The BMSCs were used as the controls for the study. According to these results, we finally selected hFOB 1.19 cells to validate the function and mechanism of hsa_circ_0001485 during osteogenic differentiation. The hFOB 1.19 is a human immortalized osteoblast cell line that expresses various osteoblast-specific markers, such as ALP, OCN, and type I collagen [44]. In vivo experiments have confirmed that hFOB 1.19 has osteogenic activity and can be used to study the proliferation and differentiation of normal human osteoblasts and the related cytokines [45, 46].

High expression of ALP activity is an early sign of osteoblast differentiation and maturation. Increased ALP activity enhances bone formation and promotes the formation of bone matrix mineralization [47]. As an essential transcription factor for osteoblast differentiation, RUNX2 encourages the expression of osteoblast secretion proteins, OCN and OPN [48]. After inhibiting hsa_circ_0001485 with small interfering RNA, we found that the ALP enzyme activity in human osteoblast hFOB 1.19 cells was significantly reduced, and the number of calcified nodules was also reduced. Simultaneously, RUNX2, OCN, and OPN levels were also reduced. The overexpression of hsa_circ_0001485 in the osteogenic hFOB 1.19 cells enhanced osteogenic differentiation. However, circRNAs, such as circ-ITCH, circ_0006766, and circ_0000020, among others, have been proven to promote the osteogenic differentiation of BMSCs [49–51]. Our research highlighted that in osteoblast hFOB 1.19 cells, overexpression of hsa_circ_0001485 improved osteoblast differentiation, which was undoubtedly a good therapeutic direction for treating OP.

The TGFβ-BMP signaling is vital in embryonic bone development and postnatal bone homeostasis [52]. The TGF-βs and BMPs act on the receptor complex and transduce signals to classical Smad-dependent signaling pathways (TGF-β/BMP ligands, receptors, and Smads), and nonclassical Smad-independent signaling pathways (p38 mitogen-activated protein kinase/p38 MAPK) regulate the differentiation of mesenchymal stem cells during bone development, formation, and homeostasis [53, 54]. The TGFβ binds to the TGFβ receptor complex to phosphorylate Smad2/3 and regulates cell functions, while the BMPs bind to the BMP receptor complex to phosphorylate Smad1/5/8 and regulate cell functions, including cell differentiation and growth [55]. Our MS data showed that 523 proteins, such as BMPR2, RRAS, FKBP1A, MAPK3, and MAPK1, are bound to hsa_circ_0001485 and were related to the TGFβ signaling pathway. The BMPR2 was selected for follow-up study as an important receptor in the BMP family, which could promote the binding and enucleation of Smad1/5/9 and Smad4 by phosphorylation, thus enabling osteogenic differentiation and angiogenesis and inhibiting lipid synthesis [56]. In nontraumatic osteonecrosis of the femoral head, miR-100-5p inhibited the BMPR2-Smad1/5/9 signaling pathway by reducing the expression of BMPR2 and destroying the bone formation of hBMSCs [56]. In human dental pulp stem cells, overexpression of circRFWD2 activated the BMP-Smad pathway through the BMPR2 receptor and targeted miR-6817-5p to enhance osteogenic differentiation [57]. The literature review shows no reports on the regulatory relationship between hsa_circ_0001485 and BMPR2. In our research, inhibition of BMPR2 by small interfering RNA and inhibitor significantly inhibited osteogenic differentiation induced by overexpression of hsa_circ_0001485. This occurrence suggested that overexpression of hsa_circ_0001485 activated the BMP-Smad signaling pathway by targeting the binding of BMPR2, thereby promoting the protein levels of RUNX2, OPN, and OCN and enhancing the osteogenic differentiation of the osteogenic hFOB 1.19 cells. Besides, we discovered that hsa_circ_0001485 can only upregulate BMPR2 protein level but has no effect on mRNA level. Recently, it is seen that circRNAs can function by interacting with proteins [58]. Thus, the circRNA-protein interaction modes mainly include altering protein interactions, tethering or isolating proteins, recruiting proteins to chromatin, forming circular RNA–protein–mRNA complexes, and protein translocation or redistribution [59]. In comparison, the specific mechanisms of hsa_circ_0001485 and BMPR2 protein have not been explored, which is also one of this study’s limitations. In future studies, we will investigate further the regulatory relationship between hsa_circ_0001485 and BMPR2 protein.

Currently, the study of OP-related circRNAs is still preliminary due to limitations in bioinformatics, transcriptomics, and proteomics, the small sample size of population studies, inconsistent findings, and lack of corresponding in vivo experimental validation. Additionally, our study was conducted to investigate the possible molecular mechanisms of OP treatment with BMSCs. Studies proved that transplantation of BMSCs, as a new approach, has great potential and application that can not only bypass the side effects of drug therapy but also treat OP at its root. However, circRNA is stable and not easily degraded; therefore, it can be applied as a target molecule to treat OP. Therefore, we speculate that BMSCs transplantation modified by the hsa_circ_0001485 may have potential therapeutic implications for OP in the elderly, which is the focal path of our future research.

## Conclusion

Using RNA sequencing in this study, we identified has_circ_0001485 as closely related to osteogenic differentiation. This finding confirms that has_circ_0001485 binds to BMPR2 to activate the TGF-BMP signaling pathway, thereby promoting osteogenic differentiation of osteogenic hFOB 1.19 cells. Targeting the expression of has_circ_0001485 by small molecule drugs may play a crucial role in bone formation, providing a new strategy for improving OP bone loss.

## Data Availability

The datasets used and/or analyzed during the current study are available from the corresponding author on reasonable request.

## Abbreviations

circRNAs: Circular RNAs
OP: Osteoporosis
circRNA: Circular RNA
OPN: Osteopontin
ALP: Alkaline phosphatase
OCN: Osteocalcin
BMD: Bone mineral density
FBS: Fetal bovine serum
OE-circ1485: Overexpressihashsa_circ_0001485
qPCR: Quantitative real-time polymerase chain reaction
ALP: Alkaline phosphatase
PVDF: Polyvinylidene fluoride
MS: Mass spectrometry
AR: Alizarin red
ALR: Alkaline phosphatase
MS: Mass spectrometry
